# A Private Patients' Hospital

**Published:** 1924-06

**Authors:** 


### A PRIVATE PATIENTS' HOSPITAL.

The Duke of Connaught spent a busy day at Bath on May 16, when he opened the Private Patients' Hospital at Combe Park and the Children's Orthopædic Hospital and paid a flying visit to the Ministry of Pensions Hospital. The Private Patients' Hospital is, it is believed, the first hospital to be built solely for the accommodation of patients of moderate means, and the Duke in his speech stated that it would take 72 patients, having 24 single rooms, and four wards with 12 beds each. The hospital, he said, was an extension of the Royal United Hospital, Bath, but patients might choose their own doctors, who need not necessarily be attached to the mother hospital. The total cost of the hospital was £43,000, and the whole of it had been subscribed locally, save for a grant from the St. John Ambulance and British Red Cross Funds. Referring to the treatment to be given at the Orthopædic Hospital, His Royal Highness spoke of it as " one of the greatest achievements of modern hospital work." The hospital is to be the centre of orthopædic work for the adjoining counties, and there will be several out-stations and clinics for after-care. These will be visited by the local doctors who have previously had the patients under their care. The hospital is a black-and-white bungalow, and sixteen little patients were lying out in their red-covered beds in the sunshine. The Duke gave enormous pleasure to the children by speaking to each one of them, inquiring about their dolls, asking their names and generally taking a personal interest in them. At the Ministry of Pensions Hospital, too, he made a special point of taking to the patients, and his visit must have been a very bright spot in their monotonous lives.

